# Decoding of the speech envelope from EEG using the VLAAI deep neural network

**DOI:** 10.1038/s41598-022-27332-2

**Published:** 2023-01-16

**Authors:** Bernd Accou, Jonas Vanthornhout, Hugo Van hamme, Tom Francart

**Affiliations:** 1 ExpORL, Department of Neurosciences, KU Leuven, Leuven, Belgium; 2 PSI, Department of Electrical Engineering, KU Leuven, Leuven, Belgium

**Keywords:** Biomedical engineering, Electroencephalography - EEG, Neurology

## Abstract

To investigate the processing of speech in the brain, commonly simple linear models are used to establish a relationship between brain signals and speech features. However, these linear models are ill-equipped to model a highly-dynamic, complex non-linear system like the brain, and they often require a substantial amount of subject-specific training data. This work introduces a novel speech decoder architecture: the Very Large Augmented Auditory Inference (VLAAI) network. The VLAAI network outperformed state-of-the-art subject-independent models (median Pearson correlation of 0.19, *p* < 0.001), yielding an increase over the well-established linear model by 52%. Using ablation techniques, we identified the relative importance of each part of the VLAAI network and found that the non-linear components and output context module influenced model performance the most (10% relative performance increase). Subsequently, the VLAAI network was evaluated on a holdout dataset of 26 subjects and a publicly available unseen dataset to test generalization for unseen subjects and stimuli. No significant difference was found between the default test and the holdout subjects, and between the default test set and the public dataset. The VLAAI network also significantly outperformed all baseline models on the public dataset. We evaluated the effect of training set size by training the VLAAI network on data from 1 up to 80 subjects and evaluated on 26 holdout subjects, revealing a relationship following a hyperbolic tangent function between the number of subjects in the training set and the performance on unseen subjects. Finally, the subject-independent VLAAI network was finetuned for 26 holdout subjects to obtain subject-specific VLAAI models. With 5 minutes of data or more, a significant performance improvement was found, up to 34% (from 0.18 to 0.25 median Pearson correlation) with regards to the subject-independent VLAAI network.

## Introduction

In recent literature, neural tracking of speech has been investigated across different invasive (e.g., ECoG^1^ and sEEG^2^) and non-invasive modalities (e.g., fNIRS^3^, MEG^4^, and EEG^5,6^). Better results have been obtained with invasive methods due to their better spatial resolution and signal-to-noise ratio (mainly due to the absence of the attenuation of the skull and skin) compared to non-invasive methods. However, non-invasive methods have broader application potential (e.g. for clinical use) and can be relatively cheap (in the case of electroencephalography (EEG)). Different methodologies have been developed to detect the neural tracking of speech, e.g. by decoding speech from brain signals^5–7^, or by translating both brain signal and speech features to a similar representation^8,9^. Neural tracking in EEG has been found for multiple acoustic representations of speech, such as the spectrogram^7,10^ or envelope representations^5,6,11,12^. Additionally, neural tracking has been shown for higher order representations such as semantic dissimilarity, cohort entropy, word surprisal, and phoneme surprisal^13–16^. Diagnostic tests can be developed that exploit the neural tracking of these features^17^. The speech envelope, for example, has been successfully linked to speech understanding^6,12,18^, and atypical phonological tracking has also been linked to dyslexia^19^.

Most commonly, linear models are used^5–7,20^. Unfortunately, the reconstruction scores are low (correlation of 0.1-0.2 between actual and reconstructed envelope for subject-specific linear EEG decoders, 0.05 for subject-specific linear forward models) with high inter-subject variability. Subject-independent models trained on a separate dataset of other subjects would be preferable as no training data for the model has to be collected^12^, but reconstruction scores are even lower for linear models in the subject-independent setting, rendering them less useful for analysis than their subject-specific counterparts^21^.

Deep, non-linear artificial neural networks have been proposed as an alternative over linear models to model the complex non-linear brain^9,11,22–24^. Recently, deep learning methods have been successfully applied to the match/mismatch paradigm for EEG data^9,11^. In this paradigm, a (non-)linear transformation of the EEG is compared to a (non-)linear transformation of a time-aligned/matched stimulus segment and a non-time-aligned/mismatched segment. The task of the model is then to identify which of the two proposed stimulus segments was time-aligned with the EEG. This method has been successfully linked to speech intelligibility^12^. Following recent advances in the match-mismatch paradigm, Thornton et al.^21^ have also shown improvements in decoding performance using neural networks in subject-specific and subject-independent settings. While deep learning is a popular method to learn complex patterns from considerable amounts of data, the low signal-to-noise ratio for auditory EEG (−10 to −20dB SNR) poses significant challenges.

We present a new decoding neural network named the *Very Large Augmented Auditory Inference* (VLAAI) network, which improves decoding performance far beyond linear methods and beyond the results of Thornton et al.^21^ (*p*<0.001).

## Results

We compared the VLAAI network to previously published state-of-the-art subject-independent models in the Comparison with baselines subsection: a linear decoder, the CNN (a convolutional neural network based on EEGNET^25^) and FCNN (a multilayer perceptron based on De Taillez et al.^22^) of Thornton et al.^21^. All models reconstructed the stimulus speech envelope from EEG across subjects. We performed an ablation study to identify which parts of the VLAAI network are responsible for what part of the decoding performance (see the Ablation Study subsection), followed by a series of experiments to test generalization (see the Generalization and Influence of amount of subjects/data seen during training subsections) and subject-specific finetuning (see the Finetuning subsection).

### VLAAI

**Figure 1:**
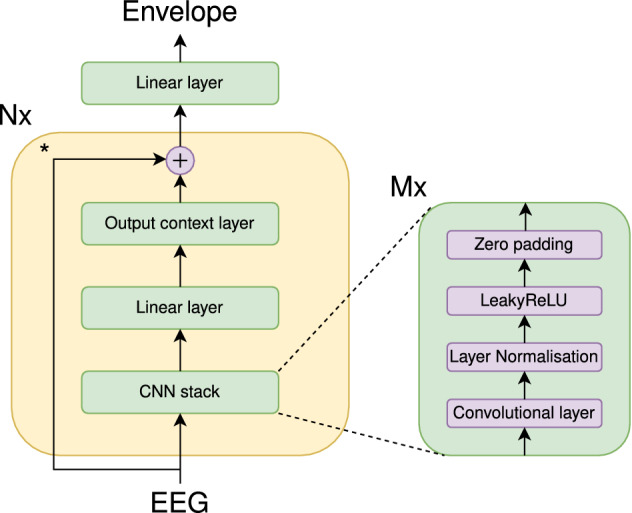
Structure of the proposed VLAAI network. The asterisk next to the skip connection indicates that it is not present in the last repetition of that block.

We propose a new model, called the VLAAI network, which consists of multiple (*N*) blocks, each consisting of 3 different parts (see also Fig. 1). The first part is the CNN stack, a convolutional neural network. This convolutional neural network consists of *M* = 5 convolutional layers. The first three layers have 256 filters, while the last two layers have 128 filters. Layer normalization^26^, a LeakyReLU^27^ activation function, and zero-padding with 7 samples at the end of the sequence are applied after every layer. The zero-padding ensures that the time dimension of the output matches that of the input, which is necessary to be able to apply skip connections later on. The second part is a simple, fully connected layer of 64 units, which recombines the output filters of the CNN stack. The last part is the output context layer. The function of this layer is to integrate predictions for previous timesteps to enhance the prediction for the current timestep. This can be viewed as an internal smoothing step: in theory, the model can leverage the previous prediction context into account to correct or enhance an unlikely prediction for the current timestep. The output context layer is implemented as a convolutional layer with a kernel of 32 and 64 filters, combined with a LeakyReLU^27^ non-linearity and layer normalization^26^. By padding the front of the input of this layer with 31 zeros, we obtain an operation that can non-linearly transform the previous 31 samples and the current sample. At the end of each block except the last, a skip connection^28^ is present with the original EEG input. After the last block, the linear layer at the top of the VLAAI model combines the 64 filters of the output context layer into a single speech envelope.

Studies often report an integration window or receptive field^6,11^, i.e., the range of input samples that was processed to produce a single output sample. The maximal receptive field (in the format $(samples\, from\, the\, past, samples\, from\, the\, future)$) can be calculated for the VLAAI model using the following equations:

$$\begin{aligned} Maximal\, receptive\, field= & {} (N*RC_{B[0]} , N*RC_{B[1]}) \\ RC_B= & {} (-(OC - 1), (K-1)*M) \end{aligned}$$

$RC_B$ is the maximal receptive field of one block, *OC* is the output context in samples of the output context layer, and *K* is the kernel size of the CNN stack. For the default VLAAI model (*N* = 4, *M* = 5, *K* = 8 and *OC* = 32), this gives a maximal receptive field of (−124, 140) samples, corresponding to (−1.94, 2.19) seconds. Note that this is the maximal receptive field; for samples at the beginning or end of a segment, this receptive field will be smaller (e.g. for the first sample of a segment, the effective receptive field will be (0, 140) samples as no prior samples/information is present).

To prevent overfitting, weight sharing across blocks is implemented for the CNN stack and output context layer. The VLAAI network was trained with Adam using a learning rate of $10^{-3}$, with negative Pearson r as a loss function. Early stopping was applied with a patience of 5 and a minimum delta of $10^{-4}$. The batch size used during training was 64.

Code for the VLAAI network and pre-trained models can be found at https://github.com/exporl/vlaai.

### Comparison with baselines

The models described in the Baseline models subsection and the VLAAI model are trained with the training set of the single-speaker stories dataset. The single-speaker stories dataset consists of 80 subjects listening to 1 hour and 46 minutes of continuous natural speech on average (approximately 141 hours in total, see also the Dataset subsection). Subsequently, the models are evaluated on the test set of the single-speaker stories dataset. This test set contains the same subjects as the training set but for unseen stimuli segments. The resulting reconstruction scores were averaged across stimuli per subject. We compared model results using a Wilcoxon signed-rank test with Holm-Bonferroni correction.

**Figure 2:**
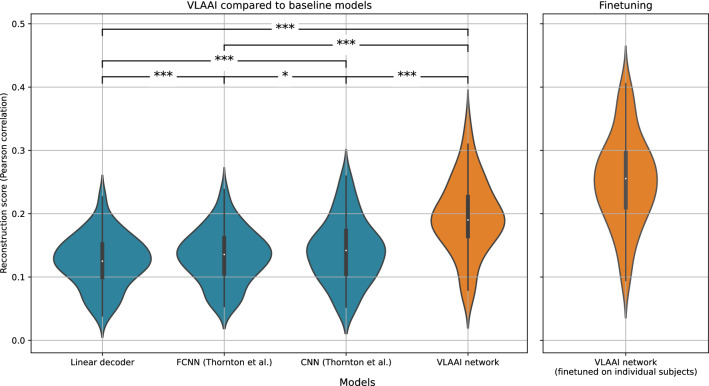
Left: Comparison of the VLAAI network with the baseline models: a subject-independent linear model, and the FCNN and CNN models presented by Thornton et al.^21^. All models were trained on data from all subjects in the single-speaker stories dataset. Each point in the violin plot is the reconstruction score for a subject (80 subjects in total), averaged across stimuli. The FCNN, CNN^21^ and VLAAI network significantly outperforms the linear decoder baseline (*p* <0.001). The CNN significantly outperforms the FCNN model $({\hbox {p}}\,=\,0.02)$. The VLAAI network significantly outperforms all baseline models ($p<0.001$), a relative improvement of 52% compared to the linear decoder. (n.s.: *p* $\ge$ 0.05, *: 0.01 $\le$ *p* < 0.05, **: 0.001 $\le$ *p* < 0.01, ***: *p* < 0.001). Right: A subject-independent VLAAI model is finetuned on data of individual subjects, resulting in one subject-specific VLAAI model per subject. The same finetuning procedure as in the Finetuning subsection is followed. The training set of the single-speaker stories dataset was used to train and finetune the subject-independent model. The test set remains unseen during training/finetuning. Each point in the violin plot is the reconstruction score for a subject (80 subjects in total), averaged across stimuli.

Figure 2 displays the resulting reconstruction scores. The FCNN, CNN^21^ and VLAAI network significantly outperformed the linear decoder baseline (*p* <0.001). The CNN significantly outperformed the FCNN model (*p* = 0.02). This contradicts the findings of Thornton et al.^21^, who found no significant difference between the FCNN and CNN models. A possible explanation is that the larger size single-speaker stories dataset can reveal differences that were hidden due to the smaller size of their dataset. Another explanation could be that the random search resulted in hyperparameters for the FCNN that are slightly suboptimal compared the hyperparameters of the CNN, although a similar random search procedure was followed as in Thornton et al.^21^ (see the Baseline models subsection for more information about the random hyperparameter search). Finally, the lower performance of the FCNN compared to the CNN may be caused by differences in measuring paradigm and stimulus characteristics between the single-speaker stories dataset and the datasets used in Thornton et al.^21^. VLAAI significantly outperformed all baseline models (median Pearson r  =  0.19, *p*<0.001), a relative improvement of 52% compared to the state-of-the-art linear model.

### Ablation study

It is notoriously hard to understand how non-linear artificial neural networks work. We conducted an ablation study to gain insight into what parts of the model are responsible for what part of the decoding performance.

**Figure 3:**
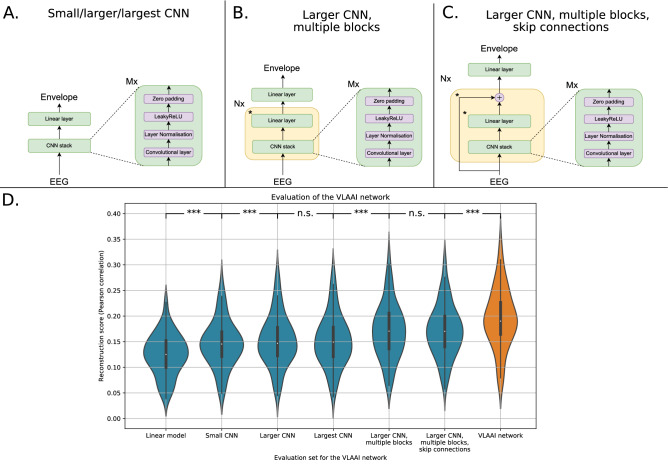
(**A**) The small/larger/largest CNN model. For the small CNN (*M*  =  2), the convolutional layers have a kernel size of 20 and 256 filters. The large CNN has five convolutional layers (*M* = 5) with 256 filters for the first three layers and 128 filters for the last two filters, all with a kernel size of 8. The largest CNN also has five convolutional layers (*M* = 5), all with 512 filters and a kernel size of 8. (**B**) The larger CNN, multiple blocks, following the structure of the larger CNN model. The asterisk next to the linear layer highlights that it is not present in the last repetition of that block. For the experiment shown in (**D**), *N* = 4 and *M* = 5. (**C**) The larger CNN, multiple blocks, with skip connections (step 5 in (**D**)). The asterisk next to the linear layer and skip connection is to highlight that it is not present in the last repetition of that block. For the experiment shown in (**D**), *N* = 4 and *M* = 5. **(D)** Ablation study of the VLAAI network. Each point in the violin plot represents a reconstruction score (Pearson correlation) for a subject, averaged across stimuli. No significant difference was found between the large and largest CNN (*p* = 0.68) and between the larger CNN with multiple blocks and the larger CNN with multiple blocks with skip connections (*p* =  0.99). The biggest increases in reconstruction score are between the linear model and the small CNN (14% increase in median reconstruction score), the larger CNN with $N=1$ and $N=4$ (10% increase in median reconstruction score) and when adding the output context layer to the penultimate model to obtain the VLAAI network (10% increase in median reconstruction score). (n.s.: *p* $\le$ 0.05, *: 0.01 $\le$ *p* < 0.05, **: 0.001 $\le$ *p* < 0.01, ***: *p* < 0.001).

Starting from the linear decoder baseline (cf the Linear decoder subsection), we added more complexity to the model in the following steps:

1. A small 2-layer (*M* = 2) CNN (kernel size 20 with 256 filters) with LeakyReLU^27^ activation function, and layer-normalization^26^ is used, as displayed in Fig. 3A.
2. The layers of the CNN are increased to 5 (*M* = 5), the same as the full VLAAI network (see also the Baseline models subsection). The CNN stack contains 256 filters for the first three convolutional layers and 128 filters for the last two convolutional layers, all with a kernel size of 8. This is the larger CNN depicted in Fig. 3A.
3. The number of filters of the convolutional layers in the CNN stack is increased to 512. This corresponds to the largest CNN as depicted in Fig. 3A.
4. The larger CNN model of step 3 is adapted to the structure displayed in Fig. 3B by increasing the number of blocks (*N*) to 4.
5. Skip connections are added to the model of to obtain the model displayed in Fig. 3C.
6. The full architecture of VLAAI is reached, as displayed in Fig. 1 (*M* = 5 and *N* = 4).

Models in subsequent steps were compared using a Wilcoxon signed-rank test with Holm-Bonferroni correction. As shown in Fig. 3D, adding more model complexity, weights and non-linearities improves performance up until step 3 (larger CNN), after which increasing the filter sizes of the model has no significant effect ($p\,=\,0.61$). Increasing the number of blocks from $N=1$ to $N=4$ delivers a big performance increase ($\approx$ a 10% increase in median reconstruction score compared to step 3). While skip connections yield no significant benefit in reconstruction score (*p* = 0.99), it has been shown that they promote stability in model training^28^. Finally, the output context layer substantially improves the performance ($\approx$ a 10% increase in median reconstruction score compared to step 5). In our experiments, $M=5$ and $N=4$ were found to be optimal for the VLAAI model.

While adding complexity is certainly beneficial to decoding performance when the models are small, it seems that at a certain size, a point of diminishing returns is reached with regard to adding weights. The increased performance added by the output context layer suggests that the model can extract beneficial information based on the previous output context.

### Generalization

Subject-independent models should be able to generalize well across different subjects, even if they are unseen in training. This generalization is crucial, as testing time is expensive.

To evaluate the across-subject generalization, the VLAAI network is trained on 80 subjects of the single-speaker stories dataset and evaluated on the test set of these 80 subjects, compared to the test set of the 26 subjects in the holdout dataset, as well as the single-speaker trials (50 seconds per trial) of the 18 subjects of the publicly available DTU dataset^29^. The results on the test set of the single-speaker stories dataset are compared to the test set of the holdout and DTU datasets.

**Figure 4:**
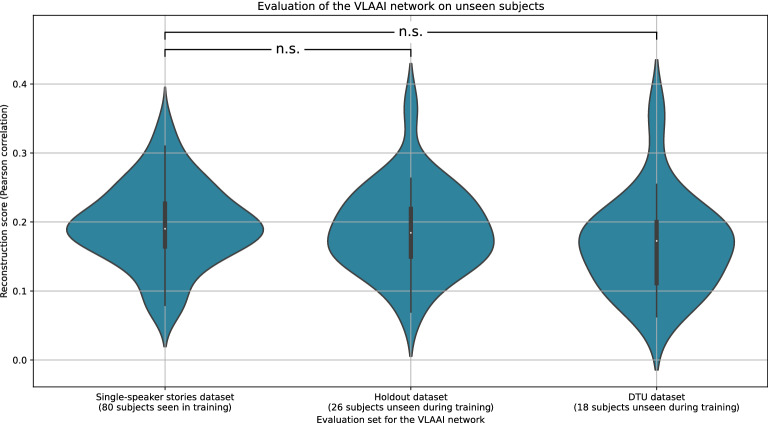
Generalization between the single-speaker stories dataset, the holdout dataset and the single-speaker trials of the publicly available DTU dataset. Each point in the violin plot is the reconstruction score for a subject, averaged across stimuli. No significant difference in reconstruction score was found between the single-speaker stories and the holdout dataset (*p* = 0.23) and between the single-speaker stories and the DTU dataset ($p=$0.08). (n.s.: *p* $\le$ 0.05, *: 0.01 $\le$ *p* < 0.05, **: 0.001 $\le$ *p* < 0.01, ***: *p* < 0.001).

**Figure 5:**
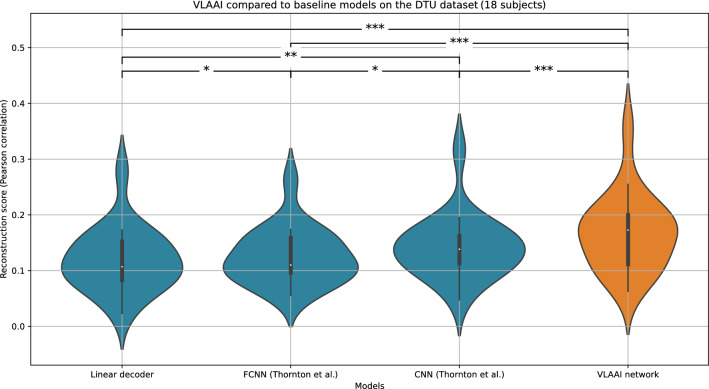
Evaluation of the baseline models and the VLAAI network on the single-speaker trials (50 seconds per trial) of the DTU dataset. While the reconstruction scores are lower for all models compared to the single-speaker stories dataset (see also Fig. 2), the general conclusions still hold. The VLAAI network significantly outperforms all other baseline models (*p*<0.01), with a relative performance increase of 62% over the linear decoder. (n.s.: *p* $\le$ 0.05, *: 0.01 $\le$ *p* < 0.05, **: 0.001 $\le$ *p* < 0.01, ***: *p* < 0.001)

In Fig. 4, the decoding performance of the VLAAI network on the test set of the 80 subjects seen during training and the test set of the 26 subjects of the holdout dataset (unseen during training) is shown. Reconstruction scores were not significantly different for the test set of the singe-speaker stories dataset compared to the test set of the holdout dataset (decrease from 0.19 to 0.18 median Pearson r, *p* = 0.23, 95% confidence interval = [−0.02, 0.04]) and compared to the test set of the DTU dataset (decrease from 0.19 to 0.17 median Pearson r, *p* = 0.08, 95% confidence interval = [−0.05, 0.06]). Additionally, to evaluate if the findings of our first experiment still hold, the baseline models (linear decoder, CNN and FCNN) of the first experiment (the Comparison with baselines subsection) and the VLAAI network were evaluated on the DTU dataset (see Fig. 5). While reconstruction scores decrease for all models compared to the Comparison with baselines subsection, the general conclusions still hold. The VLAAI network significantly outperforms all other models (*p*<0.01), with a relative performance increase of 61% over the linear decoder.

### Influence of amount of subjects/data seen during training

Subject-independent models must learn to extract subject-independent patterns and characteristics of neural tracking of the stimulus. This requires a training set with many subjects to prevent overfitting on specific characteristics of the subjects seen in training.

To assess the influence of adding data of new subjects to the training set, the VLAAI network was trained on 1–80 subjects of the single-speaker stories dataset and subsequently evaluated on the test set of the 26 subjects of the holdout dataset. A *tanh* function ($Pearson\, correlation = tanh(\frac{x}{a})^b$) is fitted on the median decoding performance of all models to characterise the relationship between decoding performance and the number of subjects used in training, using *scipy.optimize.curve_fit*^30^.

**Figure 6:**
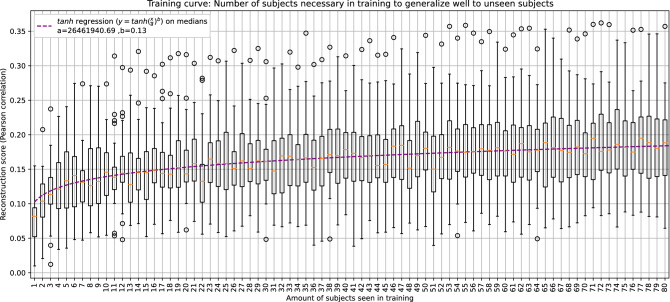
The VLAAI network was trained using 1–80 subjects of the single-speaker stories dataset and evaluated on the holdout dataset (26 subjects). Each point in the boxplot is the reconstruction score for a subject, averaged across stimuli. Median reconstruction scores increase with the number of subjects seen during training.

The results are visualized in Fig. 6. The median correlation increases with the number of subjects seen in training (80). The most dramatic increase (the 90th percentile of the increase) is seen for the first 9 subjects (from 0.08 Pearson r to 0.14 Pearson r, a relative increase of 85%). The fitted *tanh* function between the number of subjects and decoding performance can be used to extrapolate and estimate what decoding performance can be reached by including more subjects in training (e.g, approximately 3200 subjects would yield a median Pearson correlation of 0.30). Nevertheless, a plateau in decoding performance is expected to be reached when the models’ weights are saturated. The found relationship is merely indicative and could be different for other, less homogeneous datasets.

### Finetuning

In current literature, most of the focus has been on subject-specific models, as they are easy to train and well-suited for diagnostic applications. Subject-independent models can, however, be finetuned on a specific subject after subject-independent training. Starting from a previously trained subject-independent decoder can increase decoding performance as the already found general patterns are adapted to be optimal for a single subject.

The VLAAI network was trained on the training set of the single-speaker stories dataset and subsequently finetuned separately on the training set of the subjects of the holdout dataset for different amounts of training data per subject (i.e. 1 minute, 2 minutes, 5 minutes, 10 minutes, 20 minutes, 30 minutes, 1 hour and 2 hours), taken uniformly from the available recordings for each subject. To prevent overfitting, the batch size was reduced to 1, and the learning rate was lowered to $10^{-4}$. The test set of the holdout dataset was used for evaluation. To compare these finetuned subject-specific VLAAI models to the state-of-the-art for subject-specific decoding, subject-specific linear decoders were trained (with ridge regression using an integration window of 250 ms) on the training set of the holdout dataset and evaluated on the test set of the holdout dataset. The performance of subsequent models as a function of the amount of finetuning data was compared using a Wilcoxon signed-rank test with Holm-Bonferroni correction.

**Figure 7:**
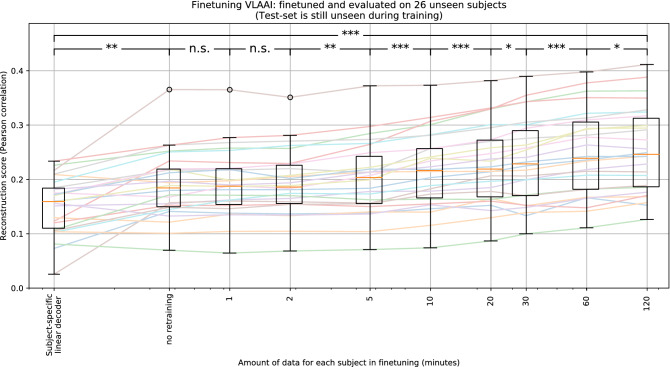
Subject-independent VLAAI, trained on the single-speaker stories dataset, finetuned on the subjects of the holdout dataset. Each point in the boxplot is the reconstruction score for a subject, averaged across stimuli. No significant increase is found between the subject-independent model (no finetuning) and models finetuned with 1 and 2 minutes of data. Starting with 5 minutes of available finetuning data, the median reconstruction score seems to increase logarithmically from 0.19 with the amount of training data to 0.25 Pearson r for 120 minutes. (n.s.: *p* $\ge$ 0.05, *: 0.01 $\le$ *p* < 0.05, **: 0.001 $\le$ *p* < 0.01, ***: *p* < 0.001).

As seen in Fig. 7, even without finetuning, the subject-independent VLAAI model already significantly outperforms the subject-specific linear decoders (median Pearson r of 0.18 vs 0.16 respectively, *p*<0.01). No significant increase is found from finetuning (the subject-independent model) with up to 2 minutes of data. The reconstruction scores of all other models were significantly different for subsequent amounts of training data (*p*<0.01). From 5 minutes onwards, the performance seems to increase logarithmically, reaching a top median reconstruction score of 0.25 Pearson r, yielding an even higher relative performance increase of 55% over the subject-specific linear decoders (*p*<0.001).

This implies that the VLAAI network can derive subject-specific patterns better suited for decoding than the already extracted subject-independent patterns, setting new state-of-the-art reconstruction scores for subject-dependent models.

## Discussion

This study introduced the new VLAAI network, compared it to baseline models from Thornton et al.^21^ and evaluated subject generalization and subject-specific finetuning. In the Comparison with baselines subsection, the VLAAI network was shown to significantly outperform the proposed baseline models (see also the Baseline models subsection). As shown by Thornton et al.^21^, increasing the number of weights does not necessarily increase decoding performance. In the Ablation Study subsection, diminished returns are indeed achieved in decoding performance when using standard non-linear artificial neural network architectures: the biggest increases in decoding performance of the VLAAI network are due to the use of a bigger model with non-linearities, the stacking of multiple blocks and the output context layer. The non-linearities might help in modelling the highly complex and non-linear auditory processing in the brain, while the output context layer can use the previous output context to refine predictions (i.e. some predictions might be implausible when taking the previous context into account). De Taillez et al.^22^ already showed the benefit of taking previous predictions into account. In their experiments, the previous context was only used to enable training with Pearson r as a loss function, while in VLAAI the effect of previous predictions is leveraged internally. The higher decoding performance of VLAAI can be used to reveal the effects of auditory processing in EEG that were previously hidden. A downside of the VLAAI network is that the model itself cannot be easily interpreted, in contrast to forward linear models that can be interpreted as temporal response functions^5,31^. However, the same is true for backward linear models, which are frequently used^32^.

In the Generalization subsection, no significant decline in reconstruction scores was found for subjects unseen during training. The DTU dataset was recorded using a similar EEG system (BioSemi ActiveTwo), but the measurement paradigm (i.e. shorter trials were presented and were part of a bigger auditory attention detection paradigm) and stimulus characteristics differed from the single-speaker stories dataset. Notably, the native language of the subject and the language of the stimuli was Danish for the DTU dataset, compared to Dutch for the single-speaker stories dataset. Despite the differences between datasets, correlation scores of the VLAAI network remain high compared to the scores of the baseline models (*p*<0.01), showing the robustness of the VLAAI model and setting a new state-of-the-art for subject-independent models.

Finetuning the VLAAI network shows that it can be transformed effectively from a subject-independent to a subject-specific model with as little as 5 minutes of additional data per subject. While the subject-independent model already significantly outperformed the subject-specific linear decoders (0.19 median Pearson r compared to 0.16 median Pearson r respectively, *p*<0.01), finetuning can increase the performance to even higher levels (median Pearson r of 0.25). This increased performance might allow uncovering previously undetectable neural processes and improve measurement efficiency, showing promise for the VLAAI network to be used in applications such as diagnostic hearing tests. In future research, better results might be obtained with even less additional data by only retraining a certain subset of layers.

Decoding other speech features might give more insight into more complex stages of the auditory system, such as the neural tracking of phonemes or semantics. However, validation on datasets of different populations (people with varying levels/causes of hearing impairment, etc.), measuring paradigms and more diverse stimuli (spontaneous speech, etc.) are still required to move towards a robust clinical application.

In conclusion, this paper proposes a new non-linear neural network architecture, which sets a new state-of-the-art in decoding the speech envelope from EEG in both a subject-independent setting (median reconstruction scores of 0.19 Pearson r, a relative improvement of 52% over the subject-independent linear decoder), and a subject-specific setting (median reconstruction scores of 0.25 Pearson r after finetuning, a relative improvement of 54% over the state-of-the-art subject-specific linear decoder models).

Our code and pre-trained models are available on https://github.com/exporl/vlaai, allowing other researchers to easily use the VLAAI network for their research/experiments.

## Materials and methods

### Baseline models

We compared the new VLAAI network to three baseline models: a linear decoder, the FCNN network and the CNN network from Thornton et al.^21^. The hyperparameters for the FCNN and CNN model were tuned on the validation set of the single-speaker stories dataset (see the Dataset subsection), following a similar procedure as Thornton et al.^21^ (Random search, 80 trials per model).

#### Linear decoder

The linear decoder reconstructs the speech envelope from EEG by using a linear transformation across all channels and a certain time/integration window. Contrary to most studies, the linear decoder here is trained subject-independently with negative Pearson r as a loss function to have a fair comparison with the other proposed subject-independent models. In preliminary experiments, Pearson r yielded better decoding performance than MSE in the subject-independent scenario. An integration window of 500 ms was used in all experiments. The linear decoder was trained using Adam^33^, using a learning rate of $10^{-3}$ on overlapping windows of 5 seconds (80% overlap) and a batch size of 64. The linear decoder was implemented in Tensorflow version 2.3.0^34^.

For the finetuning experiment (see the Finetuning subsection), subject-specific linear decoders were used. These linear decoders were trained similarly to Cross et al.^5^ and Vanthornhout et al.^6^: using ridge regression with Laplacian regularisation^35^ and an integration window of 250 ms. This was implemented using the *mne.decoding.ReceptiveField* class from MNE^36^. 15 ridge parameters were sampled logarithmically from $10^{-7}$ to $10^{7}$, and the model with the lowest validation loss was used for further analyses.

#### FCNN

The second proposed baseline is the FCNN model introduced in Thornton et al.^21^. This model is a multilayer perceptron, with weight decay applied to the hidden layers and *tanh* non-linearities, batch normalization^37^ and dropout^38^ applied subsequently. We used the same hyperparameter distributions as proposed in Thornton et al.^21^ for the random search, using early stopping with a patience of 3 and a minimum delta of $10^{-4}$, an Nadam^39^ optimizer and negative Pearson r as a loss function. After early stopping, the model with the lowest validation loss was chosen as the optimal model and used in further analyses. The optimal hyperparameters were: a learning rate of $10^{-6}$, a batch size of 256, weight decay of $10^{-6}$, a dropout rate of 35% and 2 hidden layers of 1110 and 555 nodes respectively. The model, training and evaluation code for the FCNN models were used and adapted from the author’s GitHub (https://github.com/mike-boop/mldecoders) using Pytorch 1.10.0^40^.

#### CNN

The final baseline model is the CNN model introduced in Thornton et al.^21^This model is based on the EEGNET architecture^25^. The model consists of 4 convolutional layers: a temporal convolution and a depthwise convolution^41^, which combines the channels of the temporal convolution, followed by another depthwise convolution across the time dimension. After each depthwise convolution; batch normalisation^37^, exponential linear units (ELU) non-linearities^42^, average pooling in time and spatial dropout^38^ were applied. Finally, the output of the last convolution is flattened, and all samples are combined with a fully connected layer with a linear activation. We expanded the random hyperparameter search from Thornton et al.^21^ to also search for the kernels of both average pooling operations, as our data is sampled at a different frequency (64 Hz vs 125 Hz). The kernels were sampled independently from integer values between 1 (no average pooling) and 5. The CNN was trained using the Nadam optimizer^39^ with negative Pearson r as a loss function and early stopping with a patience of 3 and a minimum delta of $10^{-4}$. After early stopping, the model with the lowest validation loss was used for further analyses. The optimal hyperparameters were: a learning rate of 0.001, weight decay of $10^{-7}$, a dropout rate of 6%, 4 for F1, 8 for D, 32 for F2 and 2 for the kernels of both average pooling operations. As with the FCNN model, code for the CNN model architecture, training and evaluation procedures were adapted from the author’s GitHub (https://github.com/mike-boop/mldecoders).

### Dataset

Our own dataset (the single-speaker stories dataset) is used to evaluate the VLAAI network and the baseline models. A subset of the publicly available DTU dataset is also used to extensively evaluate the generalizability of VLAAI and the baseline models. The single-speaker stories dataset contains 106 normal-hearing participants between 18 and 30 years old. Participants signed informed consent for this study, approved by the Medical Ethics Committee UZ KU Leuven/Research (KU Leuven, Belgium) with reference S57102. All data was collected and all experiments were performed in accordance with relevant guidelines and regulations.

Firstly, participants were asked to fill in a questionnaire, confirming that they have no neurological or auditory conditions. Secondly, the participants’ hearing was tested using a pure-tone audiogram and a Flemish MATRIX test. Participants with hearing thresholds of >30dBHL were excluded. Following the screening procedure, the EEG of participants was measured while they listened to 2-8 (on average 6) single-speaker stories. Longer stories were partitioned into multiple parts. Each part was approximately 15 minutes long. 2 out of the 10 parts were presented with speech-weighted noise at 4dB SNR, but were excluded from this study. Participants were notified before listening that they had to answer a question about the content of the story after listening as an incentive to pay close attention to the contents of the story. Throughout the recording session, participants were given short breaks. A subset of this dataset was also used in Accou et al.^11,12^, Monesi et al.^9,10^ and Bollens et al.^24^, and is available for the Auditory EEG decoding challenge (https://exporl.github.io/auditory-eeg-challenge-2023/). This dataset contains approximately 188 hours of EEG recordings (on average 1 hour and 46 minutes per subject) in total. Data from 26 (randomly chosen) subjects was designated as holdout data (the holdout dataset), while data from the remaining 80 subjects were used as standard training-, validation- and test set (the single-speaker stories dataset). The holdout dataset contains 46 hours of EEG recordings, while the single-speaker stories dataset contains 142 hours of EEG data ( 1 hour and 46 minutes of speech on average for both datasets). EEG data were collected at a sampling rate of 8192 Hz using a BioSemi ActiveTwo setup (Amsterdam, Netherlands). Electromagnetically shielded ER3A insert phones, an RME Multiface II sound card (Haimhausen, Germany), and a computer running APEX^43^ were used for stimulation. The stimulation intensity of all stimuli was fixed at 62 dBA for each ear. All recordings were performed in a soundproofed and electromagnetically shielded booth.

The DTU dataset^29^ contains EEG recordings of 18 Danish subjects that listened to natural speech in Danish spoken by 1 or 2 speakers in different reverberation settings. This dataset was also used by Fuglsang et al.^44^ and Wong et al.^45^. For our study, we used only the single-speaker trials. Each trial is approximately 50 seconds long, resulting in 500 seconds of data per subject. This data is only used for evaluation, not for training.

### Preprocessing

EEG data was high-pass filtered with a 1st order Butterworth filter with a cut-off frequency of 0.5Hz using zero-phase filtering by filtering the data in both the forward and backward direction. The speech envelope was estimated using a gammatone filterbank^46,47^ with 28 filters spaced by equivalent rectangular bandwidth with center frequencies of 50 Hz to 5 kHz. Subsequently, the absolute value of each sample in the filters was taken, followed by exponentiation with 0.6. Finally, the mean of all filters was calculated to obtain the speech stimulus envelope^48^. After downsampling EEG and speech envelopes to 1024 Hz, eyeblink artefact rejection was applied to the EEG using a multi-channel Wiener filter^49^. Next, the EEG was re-referenced to a common average. Finally, both EEG and speech envelopes were downsampled to 64 Hz.

Each EEG recording was split into a training, validation and test set, containing 80%, 10% and 10% of the recording, respectively. The validation and test set were extracted from the middle of the recording to avoid artefacts at the beginning and end of the recording. The mean and variance of each channel of EEG and the speech envelope were calculated on the training set. The EEG and envelope were then normalized by subtracting the mean from each channel and dividing by the variance for the training, validation and test set. As the DTU dataset is only used for evaluation, each trial is normalized separately and used as the test set.

All preprocessing steps were implemented in Matlab 2021a (Natick, USA), except the splitting and normalization, which were done in Python 3.6 using Numpy^50^. In all experiments, training and testing were performed on 5-second windows with 80% overlap unless specifically stated otherwise. Reported p-values for tests using Holm-Bonferroni correction for multiple comparisons are corrected p-values.

## Data Availability

The single-speaker stories dataset analyzed during the current study is available from the corresponding author on reasonable request and in compliance with the participant’s consent. The DTU dataset analyzed during the current study is available in the Zenodo repository, https://doi.org/10.5281/zenodo.1199011. A subset of single-speaker stories is available at the Auditory EEG decoding challenge (https://exporl.github.io/auditory-eeg-challenge-2023/).
